# Some of Humanity’s Debts to Vivisection*Read at the March meeting of the Medico-Legal Society, March 18, 1908.

**Published:** 1908-05

**Authors:** R. W. Shufeldt

**Affiliations:** Major Medical Department U. S. Army (ret.) Membr. Medico-Legal Society; Corr. Membr. Zool. Society of London; L’Alliance Scientifique Universelle de France; Anthropol Soc, Florence, etc., etc.


### Abstracts and Selections.

### Some of Humanity's Debts to Vivisection.*

BY R. W. SHUFELDT, M. D.,
Major Medical Department U. S. Army (ret.); Membr. Medico-Legal Society; Corr. Membr. Zool. Society of London; L'Alliance Scientifique Universelle de France; Anthropol Soc, Florence, etc., etc.

On the evening of the 19th of February (1908) the Medico-Legal Society held one of its regular meetings at the Waldorf-Astoria Hotel, upon which occasion the present writer read his paper dealing with the question of vivisection and its detractors.

*Read at the March meeting of the Medico-Legal Society, March 18, 1908.

In that communication the subject was dealt with in a very general way, the whole making too lengthy a contribution to reproduce in this journal, so, through the courtesy of the Society and Dr. Clark Bell, who presided at the above-mentioned meeting, I have been permitted to have my original paper printed elsewhere, while in the present article it is my aim to incorporate the main facts that were set forth in the paper that was read at the Waldorf-Astoria, but more particularly do I wish to elaborate those points that go to demonstrate what medical science has gained for the world and for humanity through vivisectional researches in times past, and the enormous benefits that continue to flow from such operations in the present.

It has been a matter of the greatest amount of surprise to me lately to note the names of some of the most distinguished physicians and surgeons in this country, as well as in Europe, associated with those who please to call themselves anti-vivisectionists in this old controversy, which has of late been revived on their part with all the asinine mental density of the inquisitional attacks of the enemies of science in the Fifteenth century, and it would seem with quite as much venom and vigor. If those great surgeons and physicians have said the things that the anti-vivisectionists allege that they have said against the practice, and it seems quite incredible in some instances,—why there can be but a few explanations for their attitude. They have either no knowledge of vivisection, never having enjoyed the advantage of laboratory research; or, their time being taken up with their enormous practices, they daily and unthinkingly use the facts that vivisection has given to the world, never questioning how they were obtained, by whom, or when.

Today vivisection covers a very wide field of scientific experimentation, as it not only includes experimental cutting operations upon man when justifiable, but more particularly upon various forms of animals below man,—for it likewise takes into consideration such similar experimentation, whether it be to advance our knowledge of surgery, medical practice, midwifery, the action of drugs and poisons, physiology, pathology, the relation of living beings to their environment, and other lines of research, to each and all of which it has been the main factor of advancement since vivisectional methods were first instituted.

Every profession has within it its constituency of the narrow-minded and the semi-informed. Medicine forms no exception here, and the anti-vivisectionists have drawn upon this constituency for some of its recruits. In some instances the bait of widespread notoriety was more than their light-weight brains could resist. Apart from this contingent, the main body of anti-vivisectionists is made up of sentimental women; pseudo-humanitarians; and representatives from various religious organizations, with a moderate mixture of the arch enemy to society and all other progress, the vice suppressionists.

At the outstart, I desire to say upon my own responsibility that, in the main, the representatives of the great army of doctors of medicine of the various civilized countries of the world, both living and dead, are not, nor have they been, of an ignorant class, nor of a class given to brutality, and to the performing of experiments upon living animals for the sole purpose of giving pain, and the amusement it afforded them. The sane in our community, I think, will readily admit this, whatever the anti-vivisectionists may think.

Then I desire to state, and again entirely upon my own responsibility, that the practical benefits conferred upon man and other animals which he has domesticated, through what has been learned and derived from vivisectional researches, is, in amount, absolutely incalculable. These benefits are actual and ever upon the increase. That this is true, medical evidence and medical statistics prove beyond the slightest peradventure of a doubt, and in the most incontestable manner possible.

No science can advance without continuous experimentation, and it must of necessity experiment with the materials which in the case of any particular line of scientific research it studies and deals with. In its broadest sense, the science of medicine deals with life, therefore, in its experimentation it employs such material as possesses life. The physiologist could add nothing to the knowledge of his science by experimenting with the materials used in investigation by the photographer, any more than the scientific photographer could in any way advance the acquired knowledge of his science through vivisectional research. Now, my advice to the entire body of anti-vivisectionists,—apart from those great and unthinking surgeons and physicians who have joined their ranks in the world at large, and in this country and in England in particular, is to attend to their own affairs, and let the doctors and their researches alone, for, as a matter of fact, they have no real knowledge of the necessities of either. If they really must exert their efforts to prevent cruelties to dumb animals let them turn their attention in other directions, as, for example, the cattle branding corrals on the western prairies; the ostrich farms; the restaurants, where thousands upon thousands of animals are boiled alive daily; and many similar hotbeds where unnecessary cruelties are performed, and have been performed for ages past. The examining into such operations as these falls directly and legitimately within the proper sphere of their action. Taking the sum total of the amount of pain and cruelty inflicted through the branding with a big iron brand, heated to a white heat, of thousands upon thousands of innocent little calves in this country, so far outweighs all the pain that has ever been felt by animals in the vivisectional laboratories of the world, that the latter need hardly be considered at all in comparison. And, mind you, this branding is done without the mitigation of any anesthetic, and makes only as a factor in the satisfaction of man's greed, while the facts acquired through vivisectional research make for the ultimate alleviation of suffering in both man and other animals for all time; and in all parts of the world into which the knowledge thus obtained may be extended.

Without mentioning any names then, I prefer to propound a few questions to those great and distinguished British and American surgeons and physicians, who it is alleged have joined the ranks of the anti-vivisectionists, and who, as a body subscribe to the statement that neither medicine or surgery, physiology or pathology, therapeutics or hygiene, have ever, in any way, been advanced through what we have gained by vivisectional experimentation in the broadest sense of its meaning.

These gentlemen will admit, I think, that it is of importance for us to know of the properties of the *pancreatic* juice, and the part it plays in digestion. Would it have been possible to have gained, up to the present time, such information from studies of our own species? Would the law have allowed it, and could constant demonstrations be made to medical students every year in the schools? Most emphatically, *No*. And had it not been for Bernard's brilliant vivisectional experiments upon living animals, and the forming of pancreatic fistulæ in dogs, and thus obtaining unadulterated specimens of the secretion, we would know little or nothing today of the nature and action of the pancreatic juice,—practically nothing.

It will be admitted that it is important for us to know the exact, and every action of the heart. Was not the demonstration of the shortening of the ventricles during systole made through vivisectional experiments on the lower animals, as dogs and frogs; and could this fact, so long debated, have been proved in any other way? Here we must use woorara and administer no anesthetic. How about what we know in regard to the circulation of the blood and the formation of abscesses? We would have been almost in the dark here had it not been for our examinations and experiments with the lower animals.

It is very important for us to know something of the nature of the *pressure of the blood in the arteries; the pressure in different parts of the arterial system;* the influence of respiration upon circulation; the *rapidity* of the circulation of the blood; and a great deal more referring to this most vital fluid. How have we come by nine-tenths of that information? Distinctly through vivisectional experimentation, upon living animals performed in hundreds of laboratories in various parts of the world. The experiments were of such a nature that they would never have been permitted in the case of man.

Have we learned nothing about the veinous circulation through vivisection. And how about what we know of the *respiratory* sense in the study of *respiration?* It was settled by Volkmann in 1841, and entirely through operations upon dogs; we might have waited until the crack of doom and it would never have otherwise been revealed to us.

How about Bernard's experiments upon dogs in his studies of the nature of saliva? Could these have been performed upon the human subject, and was nothing learned through vivisectional experiments in regard to these very important secretions? The physiology of *deglutition* is surely an important thing. How about the experiments of Longet upon living dogs in this matter? Half our knowledge or more is here due to vivisection. The formation of gastric fistulæ in living dogs, and the continuation of such experiments, is absolutely indispensable for the scientific study of the properties of the gastric juice and the study of the entire question of digestion. Will any sane person question for an instant what a thorough knowledge of digestion means to the medical practitioner? More than four-fifths of that knowledge has been gained through vivisectional research. So great is the bulk of this knowledge that even the importance of Dr. Beaumont's studies of the gastric juice in the case of Alexis St. Martin sink into insignificance beside it.

What would we know today of the physiological action of the intestinal juice in digestion were it not for the careful vivisectional experiments of Colin, of Frerichs, of Bidder and Schmidt, of Busch and a host of other observers in the same direction? Little or nothing. All this important physiological knowledge was accomplished through vivisection. The same may be said for our knowledge of the functions of the liver, and the action of the *bile.* Pray what would we know about such an important fluid in the economy as bile, had it not been for the hundreds of experiments that have been made by distinguished investigators upon living animals? Has the employment of biliary fistulæ in dogs brought us nothing here? Were the experiments of Schwann of no value? Could the examination of the human subject alone give us any information in regard to the properties and action of *bile?* Is the knowledge of the influence exerted by this fluid of no importance? What are the great and distinguished surgeons and physicians in the ranks of the anti-vivisectionists thinking about?

Almost our entire knowledge of the physiology of the intestines and intestinal digestion we owe to vivisectional experimentation,—and what we learned from animals in this matter was as we know, confirmed in at least one instance in the human subject by Professor Busch.

Nearly everything we know of the physiology of the spleen, kidneys and the suprarenal capsules has been acquired through our studies of these organs in the lower forms of animals. The removal of the spleen in a live dog was performed as long ago as the year 1687, and is described in the works of Malphigi, and the operation has been done hundreds of times since. We have a great deal to learn yet, with respect to the physiology of such glands as the thyroid, the thymus, and the pineal, so we can ill afford to part with such little information as we possess, much of which has come to us through vivisectional investigation, as every informed physiologist well knows.

When we come to study the literature of such a highly important subject as nutrition, we find it simply overflowing with facts that were obtained through a study of the physiology, by vivisectional means or otherwise, of the lower animals, and applying such knowledge to our own economies. The experiments of such investigators as Boussingault, M. Claude Bernard, Magendie and Bernard, who introduced thermometers into the heart of a living horse; Herwig performing the same operation on a calf; and a score or more most distinguished physiologists, having given us much that we know about animal heat. In fact more than half we know about animal heat we have obtained through experimentation upon the lower forms. Will any anti-vivisectionist question for an instant the importance of a knowledge of animal heat?

When the floor of the fourth ventricle of the brain is irritated artificial diabetes is produced, and we find sugar in the urine. The experiment is in nearly all instances made with rabbits; the operation is almost entirely painless, and is seriously interfered with if we administer an anesthetic or woorara. This experiment throws a very important light upon the glycogenic function of the liver, and I have yet to meet with a man or woman who were willing to allow me to perform the operation upon them, no matter what the knowledge meant to medicine or otherwise signified. Its value rests entirely upon a vivisectional experiment and Flint has said of it that the "production of diabetes in this way, in animals, is exceedingly interesting in its relations to certain cases of the disease in the human subject, in which the affection is traumatic and directly attributable to injury near the medulla." Many women faint when they see the instrument pushed down into the living animal's brain; anti-vivisectionists throw up their hands in holy horror; and the sentimentalists rush in to pass a bill to prohibit such brutalities. Most laymen are laboring under the impression that to cut the brain is the most painful operation that can be done on any living subject. Cutting the brain in a live person in no way influenced by an anesthetic gives rise to no pain at all. In the case of a gunshot wound of the head I have taken away a teaspoonful of brains in an unanesthetized man, and he never so much as flinched or felt it. Surely it is important to know all we can about such a disease as diabetes,—almost any one will admit that.

We owe to experiments upon living animals or those recently deprived of life nearly all we know of the physiology of the muscular system, as is shown in the well-known experiments of such able investigators as Masey, Longet, Dr. Brown-Sequard, Helmholtz and others.

In man, as in other vertebrates, there is no more important system in the economy than the nervous system, indeed, one might in truth say that it is the most important system of the body. Has our knowledge of the nervous system, in health and disease, been added to in any way through vivisectional experiments upon the lower animals? If I may be permitted to answer this question, I would say that were we to eliminate from our present knowledge of all we know of the nervous system what has been gained through the investigations upon living animals, both with and without anesthetics and woorara, of such brilliant and talented experimenters as Sir Charles Bell, Magendie, Mueller, Shaw, Waller, Bernard, Brown-Sequard, Matteuci, Chauveau, and a host of equally eminent observers since, the amount of information we would have left would be about co-equal with what physiologists knew of that system two and a half centuries or more ago. It would have been simply impossible to have gained what we here know through experiments upon human beings, and I defy anyone to demonstrate that I am wrong in this statement,—be he or she layman or laywoman, anti-vivisectionist or doctor of medicine. What we know of the nervous system through vivisection has simply relieved either in whole or in part an endless amount of pain in millions of human beings and other animals in times past, and will for all time to come. What I have just said in regard to the nervous system applies with greater or less truth to every department of surgery and medicine entailing the relief of physical and mental suffering in man and thousands of ferine and domesticated animals below him.

Space limitations will prevent me from pointing out the incalculable benefits to humanity that have flowed from vivisectional experimentation in the testing of drugs and poisons; in surgical researches; and in half a dozen equally important fields of my profession. This I may do upon some future occasion, but for the present I must close here.—*Advance Sheets Medico-Legal Journal, March, 1908.*

